# Evaluation of Luminex xTAG Gastrointestinal Pathogen Panel Assay for Detection of Multiple Diarrheal Pathogens in Fecal Samples in Vietnam

**DOI:** 10.1128/JCM.03321-15

**Published:** 2016-03-25

**Authors:** Vu Thuy Duong, Voong Vinh Phat, Ha Thanh Tuyen, Tran Thi Ngoc Dung, Pham Duc Trung, Pham Van Minh, Le Thi Phuong Tu, James I. Campbell, Hoang Le Phuc, Ton Thi Thanh Ha, Nguyen Minh Ngoc, Nguyen Thi Thanh Huong, Pham Thi Thanh Tam, Dang Thao Huong, Nguyen Van Xang, Nguyen Dong, Le Thi Phuong, Nguyen Van Hung, Bui Duc Phu, Tran My Phuc, Guy E. Thwaites, Lu Lan Vi, Maia A. Rabaa, Corinne N. Thompson, Stephen Baker

**Affiliations:** a The Hospital for Tropical Diseases, Wellcome Trust Major Overseas Programme, Oxford University Clinical Research Unit, Ho Chi Minh City, Vietnam; b Children's Hospital 1, Ho Chi Minh City, Vietnam; c Centre for Tropical Medicine, Nuffield Department of Clinical Medicine, Oxford University, Oxford, United Kingdom; d Children's Hospital 2, Ho Chi Minh City, Vietnam; e Khanh Hoa General Hospital, Khanh Hoa, Vietnam; f Dong Thap General Hospital, Dong Thap, Vietnam; g Dak Lak General Hospital, Dak Lak, Vietnam; h Hue Central Hospital, Thua Thien Hue, Vietnam; i Hospital for Tropical Diseases, Ho Chi Minh City, Vietnam; j The London School of Hygiene and Tropical Medicine, London, United Kingdom

## Abstract

Diarrheal disease is a complex syndrome that remains a leading cause of global childhood morbidity and mortality. The diagnosis of enteric pathogens in a timely and precise manner is important for making treatment decisions and informing public health policy, but accurate diagnosis is a major challenge in industrializing countries. Multiplex molecular diagnostic techniques may represent a significant improvement over classical approaches. We evaluated the Luminex xTAG gastrointestinal pathogen panel (GPP) assay for the detection of common enteric bacterial and viral pathogens in Vietnam. Microbiological culture and real-time PCR were used as gold standards. The tests were performed on 479 stool samples collected from people admitted to the hospital for diarrheal disease throughout Vietnam. Sensitivity and specificity were calculated for the xTAG GPP for the seven principal diarrheal etiologies. The sensitivity and specificity for the xTAG GPP were >88% for Shigella spp., Campylobacter spp., rotavirus, norovirus genotype 1/2 (GI/GII), and adenovirus compared to those of microbiological culture and/or real-time PCR. However, the specificity was low (∼60%) for Salmonella species. Additionally, a number of important pathogens that are not identified in routine hospital procedures in this setting, such as Cryptosporidium spp. and Clostridium difficile, were detected with the GPP. The use of the Luminex xTAG GPP for the detection of enteric pathogens in settings, like Vietnam, would dramatically improve the diagnostic accuracy and capacity of hospital laboratories, allowing for timely and appropriate therapy decisions and a wider understanding of the epidemiology of pathogens associated with severe diarrheal disease in low-resource settings.

## INTRODUCTION

Diarrheal disease remains a considerable public health challenge and is responsible for 0.8 million deaths and close to 90,000 disability-adjusted life years annually (1, 2), the majority of which occur in children <5 years of age in industrializing regions (3). Infectious diarrhea (caused by a pathogenic agent) can be caused by a number of different viruses, bacteria, and parasites. Rotavirus remains the most common cause of diarrhea in children <2 years of age (4, 5), although the introduction of rotavirus vaccines in many countries has led to a fall in incidence in locations where uptake has been significant (6, 7). Bacterial pathogens, such as Shigella spp., enterotoxigenic Escherichia coli (ETEC), Campylobacter spp., and Salmonella spp., norovirus, and the parasitic pathogens Giardia and Cryptosporidium spp. are also known to be present frequently in the stools of young children with diarrhea in resource-poor regions (4, 5, 8). As the etiology of diarrheal disease is diverse and complex, sensitive and specific diagnostics are required for prompt and accurate identification and treatment.

The detection of pathogens from stool specimens is challenging. In settings, such as Ho Chi Minh City (HCMC), Vietnam, hospital laboratories rarely perform routine microbiological culture, and if they do, they aim to isolate only a limited range of bacterial pathogens. Antimicrobial consumption in the community further reduces the sensitivity of microbiological culture for bacterial pathogens (9). Microscopy is used to detect occult white blood cells, red blood cells, and parasites, such as Entamoeba histolytica, Cryptosporidium spp., and Giardia lamblia; however, microscopic detection of parasites has poor sensitivity (10) and presents difficulties due to the requirement of a fresh specimen, the requirement for an experienced microscopist, and a lack of methodological standardization. In addition, real-time PCR systems are not used routinely in HCMC (or indeed across Vietnam) due to the cost, lack of equipment, and a paucity of trained staff. As such, a number of important pathogens, such as rotavirus, norovirus, pathovars of E. coli, and Clostridium difficile, are not routinely detected by procedures currently in place. The lack of appropriate diagnostics hinders not only clinical treatment decisions but also a wider understanding of the epidemiology and the public health implications of severe pediatric diarrheal disease in locations, like HCMC.

Given the limitations of traditional diagnostics, a multipanel pathogen identification system that is sensitive, specific, and easy to operate represents a significant improvement on classical techniques. The Luminex xTAG gastrointestinal pathogen panel assay (GPP) (Luminex Molecular Diagnostics, Austin, TX, USA) is a qualitative multiplex test cleared by the U.S. Food and Drug Administration (FDA) that is able to identify 19 enteric pathogens in one reaction in 6 h; however, the assay has a prohibitive cost in Vietnam of approximately $75 per sample (as of December 2015). The Luminex platform has been shown to be highly sensitive and specific for a variety of pathogens in several clinical settings globally (11–13). In this study, we aimed to evaluate the sensitivity and specificity of the xTAG GPP platform against those of conventional diagnostic techniques on clinical samples isolated from patients hospitalized with diarrheal disease in Vietnam.

## MATERIALS AND METHODS

### Clinical specimens and study procedures.

Fecal specimens were collected from 479 patients hospitalized with diarrheal disease from three different studies across Vietnam during 2009 to 2014. The hospitals included in the study were Children's Hospital 1, Children's Hospital 2, and the Hospital of Tropical Diseases in HCMC. Provincial hospitals in Dong Thap, Dak Lak, Khanh Hoa, and Thua Thien Hue also participated as part of the Vietnam Initiative on Zoonotic Infections (VIZIONS) initiative (14). Patients were admitted at the discretion of the treating clinician and were excluded from each of the studies if they had suspected or confirmed intussusception, multiple complications unrelated to diarrheal disease, or suspected antimicrobial-induced diarrhea. Diarrhea was defined as three watery or loose stools within 24 h or one episode of bloody and/or mucoid diarrhea (15). After collection in a sterile container, fresh stool samples were stored at 4°C at the sites and transported to the central study microbiology laboratory within 24 h. The specimens were tested using microbiological culture and real-time PCR and then stored in 20% glycerol solution at −80°C.

This study consisted of three components. First, we selected 172 samples with known etiologies from microbiological culture for validation of the xTAG GPP system. Second, we chose an additional 307 specimens that were negative by conventional culture methods, microscopy, and real-time PCR to test with the xTAG GPP to detect pathogens that we may not have identified through conventional methodologies. Finally, we used all of the 479 stool tested samples to evaluate the sensitivity and specificity of the xTAG GPP for seven pathogens by using culture and real-time PCR as the gold standards.

### Microbial culture and molecular detection of bacterial pathogens.

Stool specimens were cultured on blood agar, MacConkey agar, xylose-lysine-deoxycholate agar, selenite broth, and Campylobacter media (Oxoid, Basingstoke, United Kingdom) to isolate Shigella spp., Salmonella spp., and Campylobacter species. Apart from Campylobacter plates, which were incubated microaerophilically at 42°C, all media were incubated at 37°C overnight and then examined for growth, as previously described (16). Colonies with a morphology indicative of a named pathogen were subcultured onto nutrient agar for purity, and bacterial identity was confirmed by either API 20E (bioMérieux, France) or serotyping, where applicable. Specific serotypes of Shigella spp. and Salmonella spp. were identified by slide agglutination with antigen-grouping sera and monovalent antisera, and Campylobacter jejuni was differentiated from Campylobacter coli by the hippurate hydrolysis test.

All stool specimens were also screened for the presence of Shigella spp. (target, *ipaH*), Salmonella spp., and Campylobacter spp. (C. jejuni target, *hipO*; C. coli target, *glyA*) by real-time PCR using the following cycling conditions: 95°C for 15 min, 40 cycles of 95°C for 5 s, 60°C for 30 s, and 72°C for 30 s, as described previously (17, 18). Salmonella screening was performed using an in-house assay targeting the *invA* gene (forward primer, 5′-TCATCGCACCGTCAAARGA-3′; reverse primer, 5′-CGATTTGAARGCCGGTATTATT-3′; probe, 5′-FAM-ACGCTTCGCCGTTCRCGYGC-BHQ1-3′ FAM, 6-carboxyfluorescein; BHQ1, black hole quencher 1), under the following conditions: 95°C for 15 min, 45 cycles of 95°C for 5 s, and 60°C for 60 s (19).

### Molecular detection of rotavirus, norovirus GI/GII, and adenovirus.

For rotavirus and norovirus molecular testing, total RNA was extracted from fresh stool samples, reverse transcribed into cDNA, and used as the template to detect viruses by real-time PCR, as previously described (20). Rotavirus detection was performed by targeting the nonstructural protein 3 (NSP3). The norovirus primers and probes targeted the open reading frame 1 (ORF1)-ORF2 junction of norovirus genotype 1/2 (GI/GII) (21). PCR amplifications for rotavirus and norovirus GI/GII were performed using RNA Master hydrolysis probes (Roche Applied Sciences, United Kingdom) and optimized with 1.4 μl of activator on a LightCycler 480II (Roche Applied Sciences). Five microliters of RNA was mixed with a concentration of 20 μM for each primer and 10 μM probe, and thermal cycling was initiated at 61°C for 5 min for reverse transcription, 5 min at 95°C for amplification, and then by 45 cycles at 95°C for 5 s and 60°C for 45 s. Adenovirus real-time PCR amplification was performed using primers AdV-F (TCTTACAAAGTGCGC TTTACGC) and AdV-R (TTAAAGCTGGGRCCACGATC) and probe AdV-probe (Cy5-GACAACCGGGTKTTGGACATGGCCAG-BHQ3). Adenovirus DNA was mixed with a concentration of 20 μM for each primer and 10 μM probe and LightCycler 480 probes master (Roche Applied Sciences) and subjected a PCR cycle of 95°C for 5 min, followed by 45 cycles of 95°C for 5 s and 60°C for 1 min.

The assay was validated with positive controls using a previously described procedure (18).

### xTAG gastrointestinal pathogen panel testing.

The xTAG GPP assay is a multiplex assay that simultaneously detects Salmonella spp., Shigella spp., Campylobacter spp., C. difficile (toxins A and B), enterotoxigenic E. coli (ETEC) heat labile (LT) and heat stable (ST) enterotoxins, E. coli O157, Shiga toxin-producing E. coli (STEC) spp., Vibrio cholerae, Yersinia enterocolitica, fecal adenovirus 40/41, rotavirus A, norovirus GI/GII, Giardia spp., E. histolytica, and Cryptosporidium species. For nucleic acid extraction, 200 μl of each stool specimen was automatically extracted with 10 μl of xTAG MS2 (internal control) using the MagNA Pure-96 machine (Roche), according to the manufacturer's instructions. The xTAG GPP assay was performed according to the manufacturer's recommendations (Luminex Molecular Diagnostics, Austin, TX, USA).

### Statistical analysis.

The Luminex data were read by the TDAS software and imported and analyzed in Stata version 11 (College Station, TX, USA). Plots were made in R version 3.1.1 (R Foundation for Statistical Computing, Vienna, Austria) using the *ggplot2* package (22). Sensitivity and specificity were calculated separately using two gold standards: (i) microbiological culture and (ii) real-time PCR. As the specificity of the original Luminex cutoffs for Salmonella spp. was low (∼60%), we attempted to redefine the cutoffs to improve the sensitivity and specificity for the Salmonella assay. To evaluate the capacity of probes 1 and 2 in discriminating between positive and negative Salmonella infection, the area under the receiver operating characteristic (ROC) curve was computed for univariate logistic regression models (containing either probe 1 or 2 alone as a predictor) and a multivariate logistic regression model (containing both probes 1 and 2 as predictors). The optimal cut points for probe 1, probe 2, or for probes 1 and 2 combined were estimated using the Youden index method (in which the sum of sensitivity and specificity of the logistic models were maximized) (23).

### Ethical approval.

Stool samples were collected from three studies, all of which were approved by the local ethical committees and Oxford Tropical Research Ethics Committee (OxTREC) (no. 0109, 15-12, and 1045-13). Written informed consent was required for all patients or from parents/legal guardians if the patient was a child prior to enrollment into all three studies.

## RESULTS

### Baseline results.

Overall, 479 stool samples were collected from 92 adults (>15 years) and 387 children (≤15 years old) admitted to the hospital with diarrheal disease in Vietnam between 2009 and 2014. The median age of the adult patients was 50 years (interquartile range [IQR], 33 to 64 years), and 39% (36/92 patients) were male. In children, the median age was 16.5 months (IQR, 6.7 to 20 months), with 57% (221/387 patients) being male. A total of 105 samples (22%) were positive for a bacterial pathogen by microbiological culture, 268 samples (56%) were positive for a pathogen by real-time PCR, and 404 samples (84%) were positive for a pathogen by the xTAG GPP. From the 479 tested samples, the most commonly identified pathogens through the xTAG GPP platform were Salmonella (*n* = 210 [44%]), rotavirus (*n* = 121 [25%]), norovirus GII (*n* = 89 [19%]), Shigella (*n* = 91 [19%]), and Cryptosporidium (*n* = 83 [17%]). Though less common, the xTAG GPP also detected C. difficile (*n* = 45 [9%]), ETEC (*n* = 40 [8%]), Giardia (*n* = 15 [3%]), norovirus GI (*n* = 15 [3%]), E. coli O157 (*n* = 11 [2%]), Shiga toxin-producing E. coli (STEC) (*n* = 8 [2%]), and E. histolytica (*n* = 4 [1%]).

### Validation.

To initially validate the xTAG GPP, 40 stool samples positive for Shigella spp., 40 samples positive for Salmonella spp., and 30 samples positive for Campylobacter spp. by microbiological culture were retested using the xTAG GPP, as shown in Table 1. The proportion of positive agreement of xTAG GPP and microbiological culture was high, ranging from 90 to 100%. Furthermore, using stool samples, a previously detected viral pathogen by real-time PCR, the xTAG GPP, had similarly high agreement (90.5 to 93.0%) in 68 samples positive for rotavirus, 58 samples positive norovirus GI/GII, and 17 samples positive for adenovirus 40/41 infections.

**TABLE 1:** Number of positive Luminex xTAG GPP results on samples known to be positive by microbiological culture and real-time PCR

Pathogen detected by method	No. detected by culture/real-time PCR	No. detected by GPP	% positive agreement
Culture			
Shigella	40	40	100
Salmonella	40	38	95.0
Campylobacter	30	27	90.0
Real-time PCR			
Rotavirus	68	63	92.6
Norovirus GI/GII	58	54	93.1
Adenovirus	20	17	85.0
Shigella	44	44	100
Salmonella	48	45	93.8
Campylobacter	40	36	90.0

### Negative-specimen evaluation.

**FIG 1:**
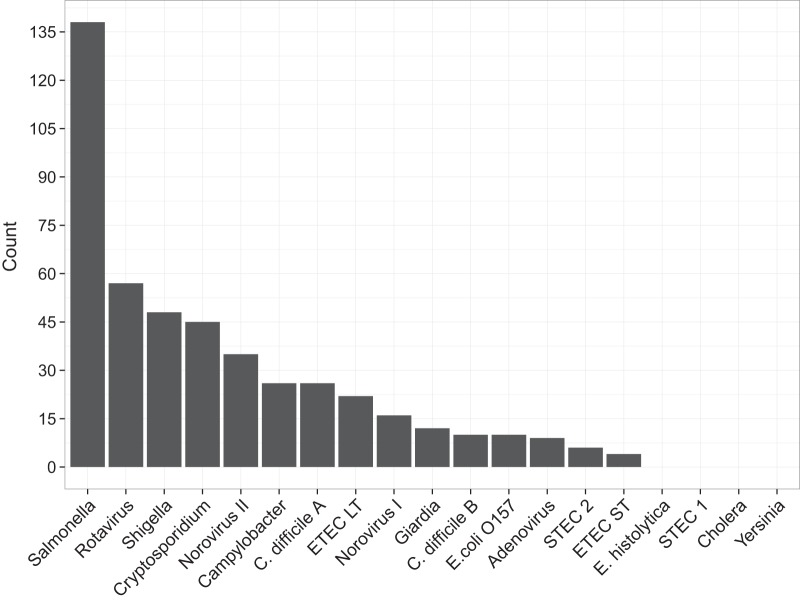
Count of pathogens detected by the Luminex xTAG gastrointestinal pathogen panel in 307 specimens that tested negative by microbiological culture and real-time PCR.

A total of 307 fecal samples that were negative by microbiological culture and real-time PCR were tested by xTAG GPP. Salmonella was the most frequently detected pathogen, present in 45% (138/307) of the initially negative specimens. Further, rotavirus and Shigella spp. were identified in 19% (57/307) and 16% (48/307) of the negative specimens, respectively. Additionally, several significant enteric pathogens that are not well studied (or even previously described in Vietnam) due to a lack of diagnostic capacity were also identified, including Cryptosporidium (15% [45/307 specimens]), C. difficile (12% [36/307 specimens]), and pathogenic E. coli (14% [42/307 specimens]), as shown in Fig. 1.

### Performance evaluation on clinical specimens.

Table 2 shows the performance of xTAG GPP in comparison to both a positive microbiological culture and a real-time PCR positive gold standard for seven enteric pathogens, including Shigella spp., Campylobacter spp., and Salmonella spp., and also rotavirus, norovirus GI/GII, and adenovirus. The xTAG GPP had high sensitivity for all pathogens, ranging from 88.2% to 100% using both culture and real-time PCR as gold standards. The specificity of xTAG GPP was also moderately high for most of the detected bacterial and viral pathogens, ranging from 88.4% to 99.3%, with the notable exception of Salmonella species. A total of 128 and 172 stool specimens were negative for Salmonella spp. by real-time PCR and culture, respectively, but were positive by the xTAG GPP assay, resulting in specificities of the xTAG GPP assay of 60.8% and 66.8% for Salmonella in comparison to real-time PCR and culture, respectively. However, when we attempted to redefine the cutoff values to improve the specificity, we found no adjusted cutoff rules that significantly improved the sum of the sensitivity and specificity for probes 1 and 2 combined compared to that of the originally defined cutoffs, according to the manufacturer's instructions.

**TABLE 2:** Sensitivity and specificity of Luminex xTAG GPP in comparison to real-time PCR and microbiological culture^*a*^

Organism detected	xTAG GPP compared to real-time PCR					xTAG GPP compared to culture				
	GPP result	No. with real-time PCR result of:		Sensitivity (% [95% CI])	Specificity (% [95% CI])	GPP result	No. with culture result of:		Sensitivity (% [95% CI])	Specificity (% [95% CI])
		Pos	Neg				Pos	Neg		
Shigella	Pos	86	6	95.6 (89.0–98.8)	98.5 (96.7–99.4)	Pos	40	51	100 (91.2–100)	88.4 (85.0–91.2)
	Neg	4	383			Neg	0	388		
Salmonella	Pos	84	128	90.3 (82.4–95.5)	66.8 (61.9–71.5)	Pos	38	172	95.0 (83.1–99.4)	60.8 (56.1–65.4)
	Neg	9	258			Neg	2	267		
Campylobacter	Pos	59	3	90.8 (81.0–96.5)	99.3 (97.9–99.8)	Pos	27	35	90.0 (73.5–97.9)	92.2 (89.3–94.5)
	Neg	6	411			Neg	3	414		
Adenovirus	Pos	23	5	92.0 (74.0–99.0)	98.9 (97.4–99.6)					
	Neg	2	449							
Norovirus GI	Pos	15	5	88.2 (63.6–98.5)	98.9 (97.5–99.6)					
	Neg	2	457							
Norovirus GII	Pos	85	4	96.6 (90.4–99.3)	99.0 (97.4–99.7)					
	Neg	3	387							
Rotavirus	Pos	117	4	92.9 (86.9–96.7)	98.9 (97.1–99.7)					
	Neg	9	349							

a GPP, gastrointestinal pathogen panel; Pos, positive; Neg, negative; 95% CI, confidence interval.

### Coinfection.

**FIG 2:**
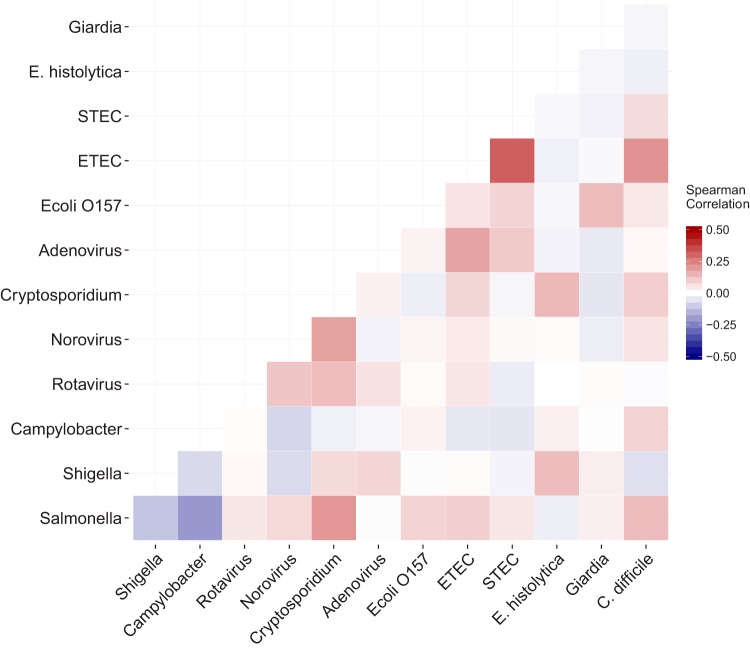
Correlation matrix of coinfections from 479 diarrheal stool samples. Common pathogens detected with the Luminex xTAG gastrointestinal pathogen panel are listed in a matrix format along the *x* and *y* axes. The color within each square represents the Spearman correlation coefficient for each pairwise coinfection. The darker the red, the more positive the correlation, and the darker the blue, the more negative the correlation. Note that the range of the color scale spans −0.5 to 0.5.

Of all evaluated stool samples with a detected pathogen, 58% (233/404 samples) had more than one enteric pathogen identified by the xTAG GPP assay. The majority of coinfections had two pathogens detected, although the number of detected pathogens was as high as seven in one sample. As shown in Fig. 2, Cryptosporidium spp. and ETEC were the most common pathogens detected in stool samples, with other pathogens, including E. histolytica, Salmonella spp., rotavirus, and norovirus with Cryptosporidium spp. and STEC, C. difficile, and adenovirus with ETEC. Additionally, norovirus and rotavirus were commonly identified in the same sample. Notably, patients with Campylobacter infections tended to have negative correlations with most other pathogens, suggesting a higher prevalence of monoinfection.

## DISCUSSION

Diarrheal disease is the second most common cause of death in children <5 years of age globally (3). The need for rapid and reliable diagnostics is important for treatment decisions and is even more pressing given the alarming trends of antimicrobial resistance in many of the most common bacterial enteric pathogens (24), which may in part be due to the unregulated and excessive use of antimicrobials worldwide. In this study, we demonstrate that the multiplex molecular Luminex xTAG GPP detection system is both highly sensitive and specific compared to both microbiological culture and real-time PCR for the majority of the surveyed pathogens. Additionally, the xTAG GPP system allowed for identification of a number of important pathogens, including ETEC and Cryptosporidium spp., which are not detected through routine procedures in hospitals in Vietnam or across other parts of Southeast Asia.

The sensitivity and specificity of the xTAG GPP were >88% for all compared pathogens (three bacteria and four viruses) using both culture and real-time PCR; however, a notable exception was Salmonella. Our sensitivity and specificity findings are similar to, if somewhat lower than, those of other studies conducted in the North America, Europe, and China (11–13, 25). The low specificity (∼60%) of Salmonella detection in our study has not commonly been reported in other studies, although Wessels et al. (26) found a higher rate of false-positive Salmonella detection in a small study from the Netherlands. This may be due to a high rate of asymptomatic carriage with Salmonella in Vietnamese children (8) or cross-reactivity with an alternative component of the gastrointestinal microbiota present in this population. The low specificity of the xTAG for detecting Salmonella likely led to an artificially inflated rate of overall coinfection in our samples (58% of positive samples) compared to that in work conducted elsewhere (13, 25, 27, 28). However, the rates of pairwise coinfections, such as C. difficile and Cryptosporidium spp., are similar to those found in a multicenter Italian study (29). An obvious issue raised by the high rate of coinfection and the use of the xTAG GPP in general is pathogenicity. Platts-Mills and colleagues (5) recently found that Cryptosporidium spp., STEC, and Shigella spp. were more commonly associated with severe diarrhea than other pathogens through a birth cohort that sampled healthy and diarrheal stools. In order to investigate relative pathogenicity in the future, use of the xTAG GPP platform in combination with a quantitative technique (30) may provide more insight into the relationship between the presence of a pathogen and clinical disease.

Through the use of the xTAG GPP platform, we were able to identify a far wider range of pathogens than those normally screened in hospitals and in research studies in Vietnam. Cryptosporidium for example, was the fifth most common pathogen detected in our sample set (17%), although almost nothing is known about the epidemiology of this parasite in Vietnam. Globally, Cryptosporidium is known to be common in young children with moderate-to-severe diarrhea and can commonly be identified at a very high rate (15 to 25%) in children with persistent diarrhea (4, 31). Because the intestinal damage due to infection with Cryptosporidium can result severe long-term sequelae (32), specific studies on this parasite and its epidemiology are warranted. Additionally, 9% of the patients in our cross-sectional sample had C. difficile detected in their stool. C. difficile is known to be much less common in children than adults, and asymptomatic infection with toxigenic strains is common (33–35) and may represent a reservoir for infection (36), although additional investigation into the epidemiology and clinical significance of C. difficile infection in children in this setting is warranted. The xTAG GPP assay allows for the detection of such important pathogens and may aid not only in treatment decisions but also in the generation of hypotheses for further epidemiological and etiological studies.

While the benefits of the xTAG GPP system are numerous, a significant drawback for use in a country, such as Vietnam, is the cost. The price of a single xTAG GPP reaction is currently prohibitive for Vietnam, although recent work from the United Kingdom has demonstrated that routine use of the multiplex diagnostic led to massive savings at the hospital level due to rapid identification of noninfectious diarrheal disease and subsequent reduction of the need for single-patient rooms that far exceeded the cost of implementing the xTAG GPP itself (37). Whether or not such economic analyses are applicable to Vietnam is questionable, and future such analyses tailored to industrializing regions are recommended. An additional drawback is the lack of an organism for antimicrobial susceptibility testing. Given the rampant antimicrobial resistance found in pathogens, such as Shigella spp., in Vietnam (8), this is a significant limitation.

Our study has several limitations. First, the microbiological culture was performed at different laboratories across Vietnam and may have introduced variability into our results. Second, we were not able to identify some pathogens, such as V. cholerae or Yersinia, so we were unable to evaluate the performance of the xTAG GPP against these pathogens, which may be more relevant in other settings across the region. Notwithstanding these limitations, our results shed valuable insight into the accuracy of the xTAG GPP system and its utility in an industrializing setting.

In conclusion, the Luminex xTAG GPP diagnostic platform is highly sensitive and specific for common bacterial and viral enteric pathogens in Vietnam, with the notable exception of Salmonella species. Furthermore, the xTAG GPP platform allowed for detection of a number of important pathogens that are not routinely identified through current hospital laboratory procedures in Vietnam. While there are a number of benefits to the xTAG GPP platform, cost and lack of an organism for antimicrobial susceptibility testing may represent barriers to widespread use in low-income regions. However, the use of such a diagnostic in locations, like HCMC, Vietnam, would dramatically improve the ability of clinicians to appropriately treat gastrointestinal infections in a timely manner, provide evidence for a more prudent use of antimicrobials, and allow for an unprecedented level of epidemiological granularity to aid in prevention and control efforts for diarrheal disease.
